# Supplementary material for: Cooperation between oncogenic Ras and wild-type p53 stimulates STAT non-cell autonomously to promote tumor radioresistance
Source: Commun Biol. 2021 Mar 19;4:374. doi: 10.1038/s42003-021-01898-5 (PMC7979758; doi:10.1038/s42003-021-01898-5)
Supplement: Supplementary file 2 — Supplementary Information [file 42003_2021_1898_MOESM2_ESM.pdf]

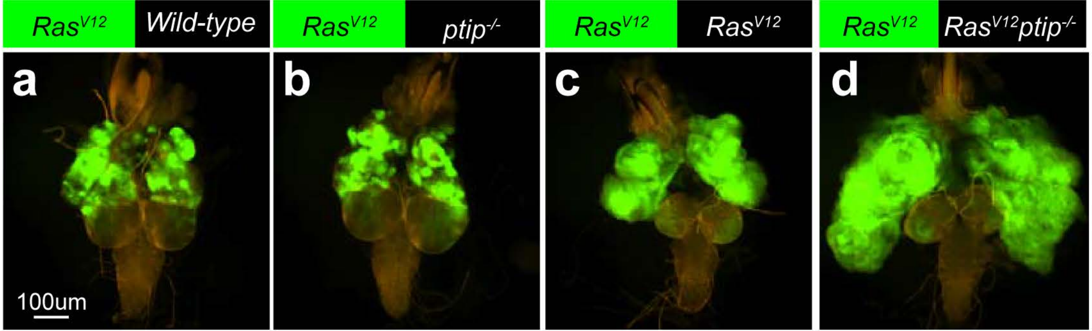

Supplementary Figure 1. Oncogenic Ras and the *ptip*$^{-/-}$ mutation cooperate to accelerate the growth of nearby *Ras*^{V12} clones.

Images showing the growth of mosaic eye imaginal tissue from dissected brain complexes. Eye imaginal tissues contain GFP-positive *Ras*^{V12} clones juxtaposed against wild-type (a) or ptip-/- (b) or *Ras*^{V12} (c) or *Ras*^{V12}*ptip*$^{-/-}$(d) mutant clones.

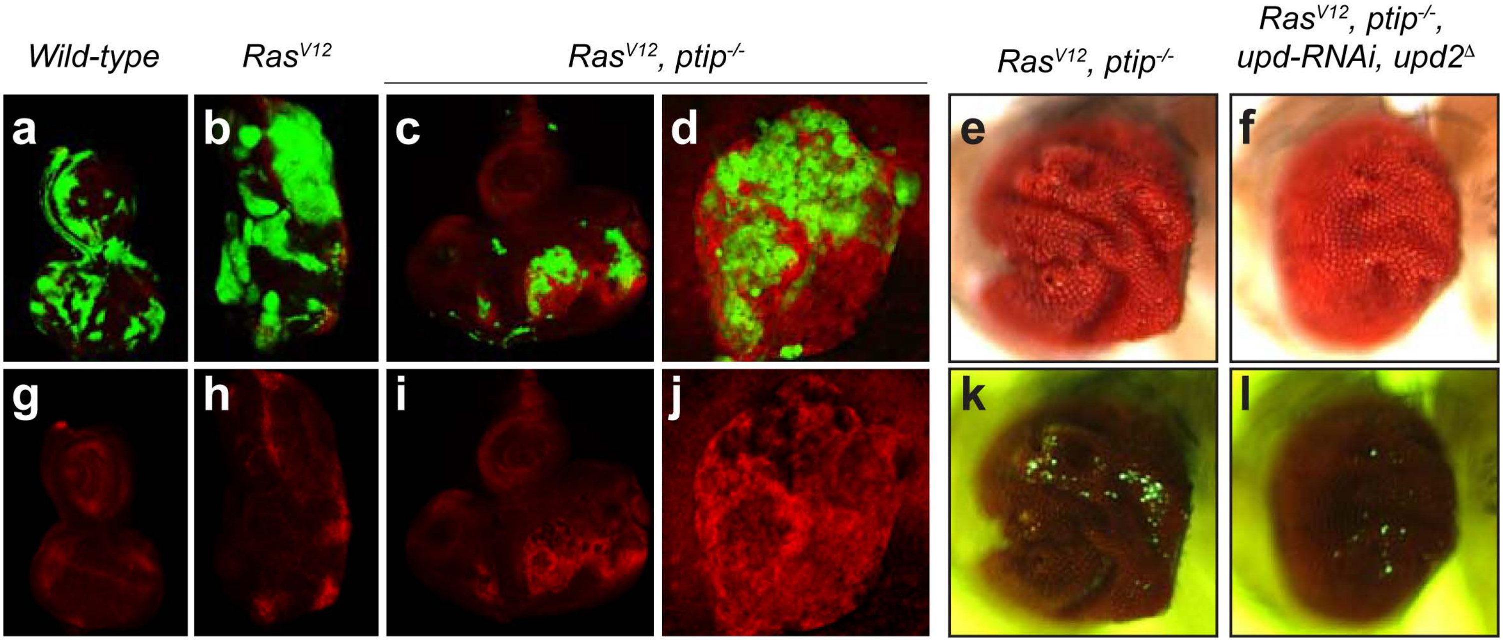

Supplementary Figure 2. Oncogenic Ras and *ptip*$^{-/-}$ stimulate nonautonomous tissue overgrowth via paracrine activation of STAT signaling.

(a-d) Representative images of dissected eye imaginal tissues containing GFP-labeled clones of wild-type (a) or *Ras*^{V12} (b) or *Ras*^{V12}*ptip*$^{-/-}$ (c-d) mutant cells stained with an antibody that detects STAT92E (red). Corresponding individual STAT92E channel images are shown in bottom panels (g-j).
(e-l) Matched light and fluorescence images of adult eyes containing Ras^{V12}ptip$^{-/-}$ mutant clones in the absence (e, k) or presence (f, l) of upd-RNAi and upd2 deletion (upd2$^{\Delta}$).

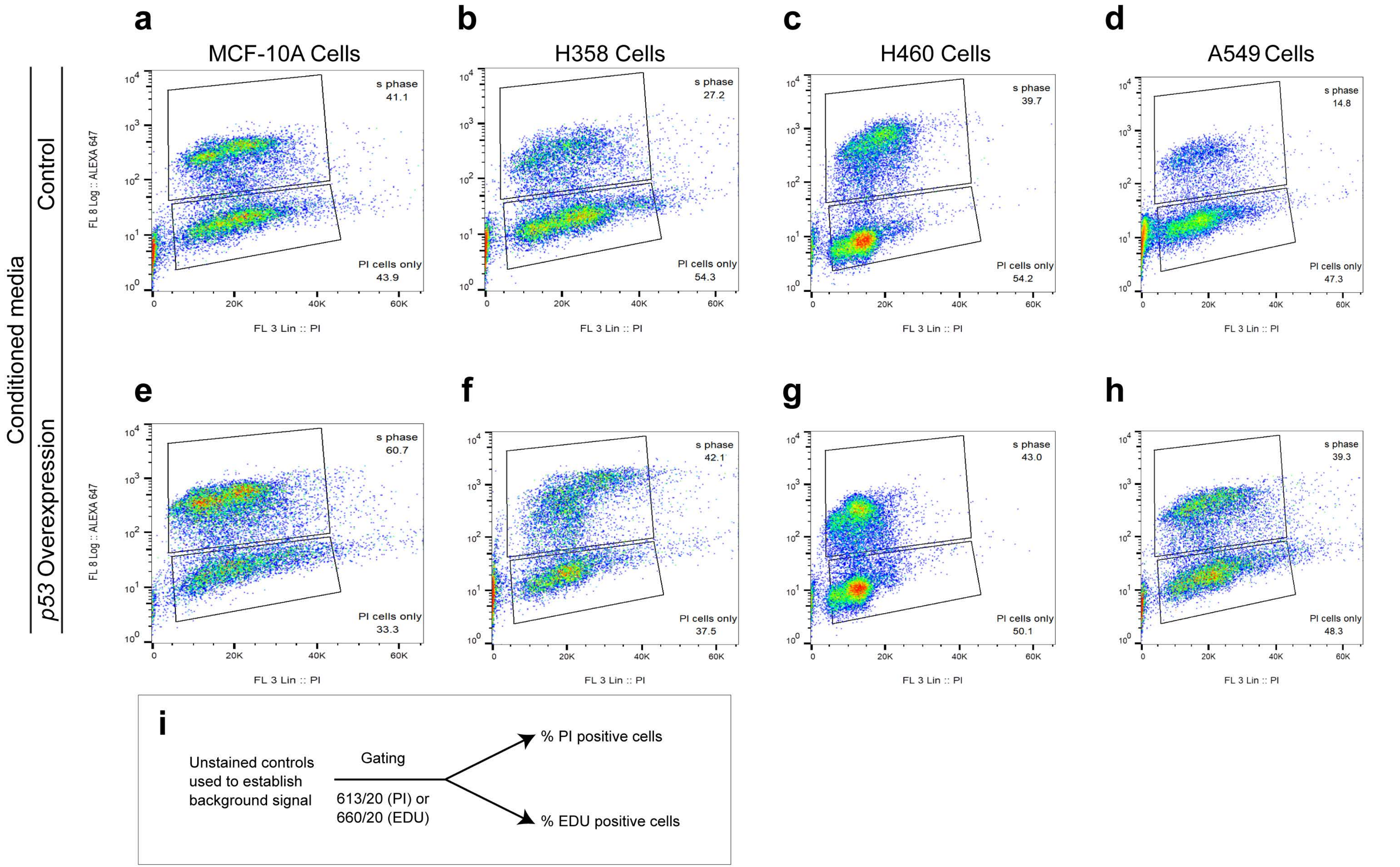

Supplementary Figure 3. Media conditioned with oncogenic Ras cells overexpressing *p53* elevate the cell proliferation potential of human cancer cells.

(a-h) images of fFlow cytometry plots from experiments using the indicated cancer cells. Cancer cells were co-labeled with EDU-647 and propidium iodide (PI) to determine the proportion of proliferating cells and dying cells, respectively. Top row (a-d) shows the proportion of proliferating and dying cells when cells are cultured in control media (conditioned with unmodified matching cells). Bottom row shows the proportion of proliferating and dying cells when cells are cultured in media conditioned with matching p53-overexpressing cells instead.
(i) Schematic of FACS gating strategy. Unstained cells were used to establish background signals in each experiment. 613/20 or 660/20 bandpass filters were subsequently applied to score the proportion of PI or EDU-647 positive cells, respectively.

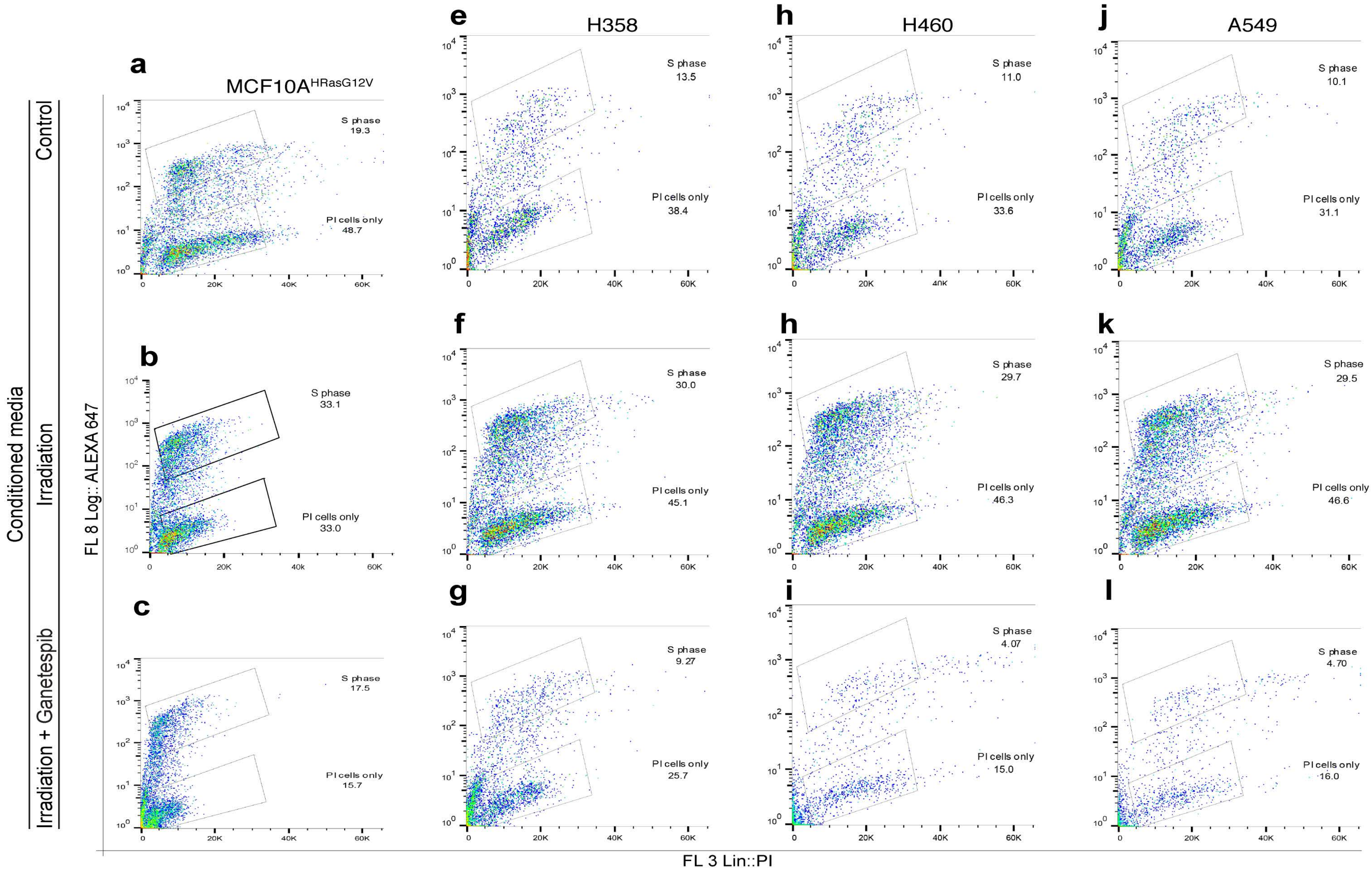

Supplementary Figure 4. Media conditioned with irradiated cells expressing oncogenic Ras elevate the cell proliferation potential of human cancer cells.

(a-l) Flow cytometry plots from experiments using the indicated cancer cells. Cancer cells were co-labeled with EDU-647 and propidium iodide (PI) to determine the proportion of proliferating cells and dying cells, respectively. The top row (a, d, g, and j) shows the proportion of proliferating and dying cells when cells are cultured in media conditioned with unmodified matching cells (baseline controls). The middle row (b, e, h, and k) shows the proportion of proliferating and dying cells when cells are cultured in media conditioned with the matching irradiated cells. The bottom row (c, f, i, and l) shows the proportion of proliferating and dying cells when cells are cultured in media conditioned with matched irradiated cells in the presence of Ganetespib.

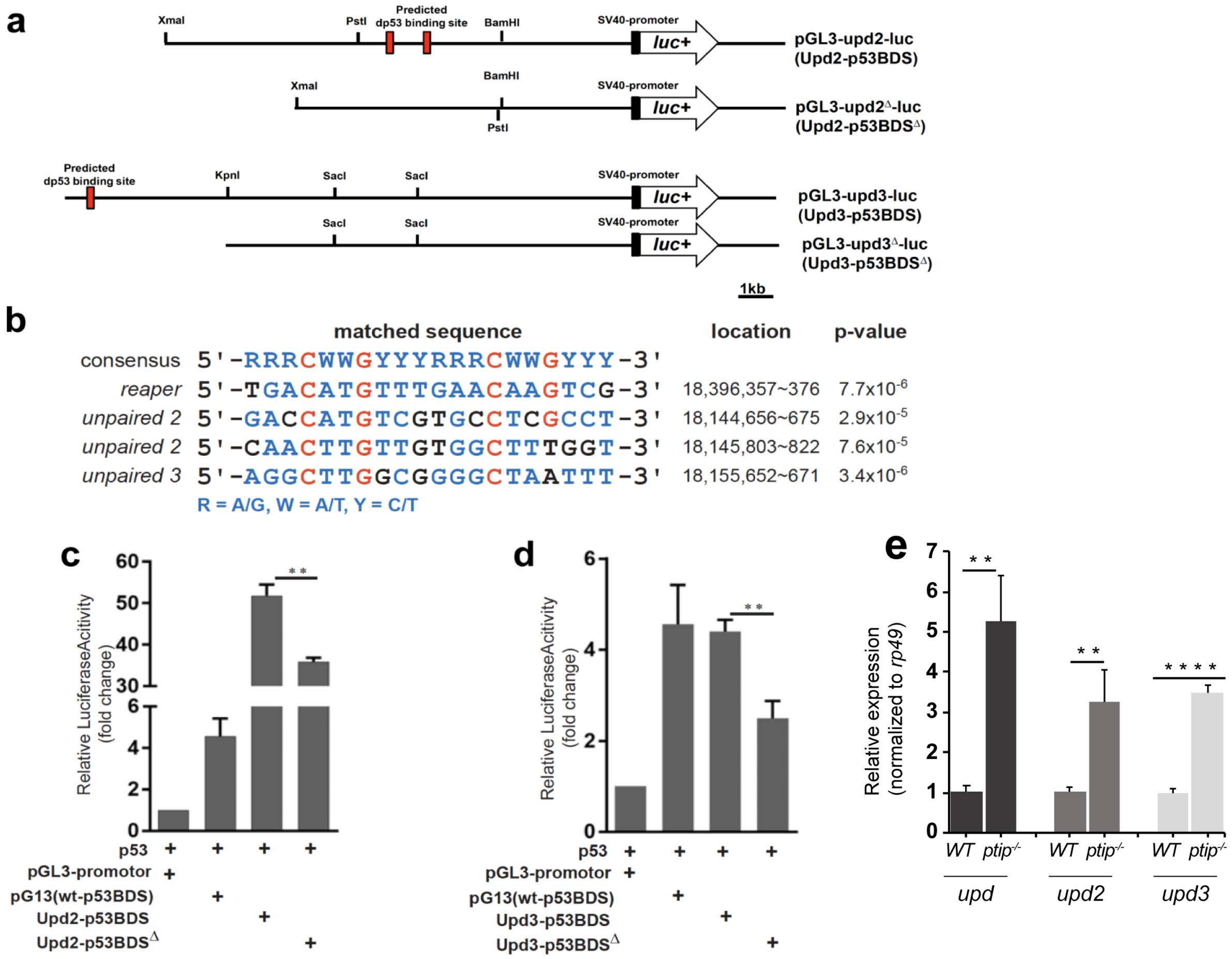

Supplementary Figure 5. In silico identification of functional p53 binding sequences upstream of unpaired genes and stimulation of upd in ptip$^{-/-}$ tissues.

(a) Graphical representation of the strategy used to develop the upd promoter activity reporter assay. Putative p53 binding sites (p53BDS) from the upstream regions of upd2 and upd3 genes are denoted with red boxes.
(b) Genomic sequences showing reaper consensus p53BDS matched to upd2 and upd 3 upstream sequences. Note that the p53BDS from upstream of reaper gene is a well-defined p53 sequence. Corresponding locations and p-values are shown to the right.
(c, d) Luciferase activity in S2R+ cells after copper sulfate induction of p53 expression in the absence or presenceof Upd2-p53BDS, Upd3-p53BDS, as well as p53BDS-deleted control (Upd2-p53BDS^{D} Upd3-p53BDS^{D}) sequences. T-test significance levels are (*) p<0.05 and (**) p<0.01.
(e) qPCR data showing relative expression of upd1 or upd2 or upd3 in wild type versus ptip-/- eye imaginal disc.
Expression was normalized to the transcript levels of the housekeeping gene rp49. T- test significance levels are (**) p=0.01, (***) p=0.003.

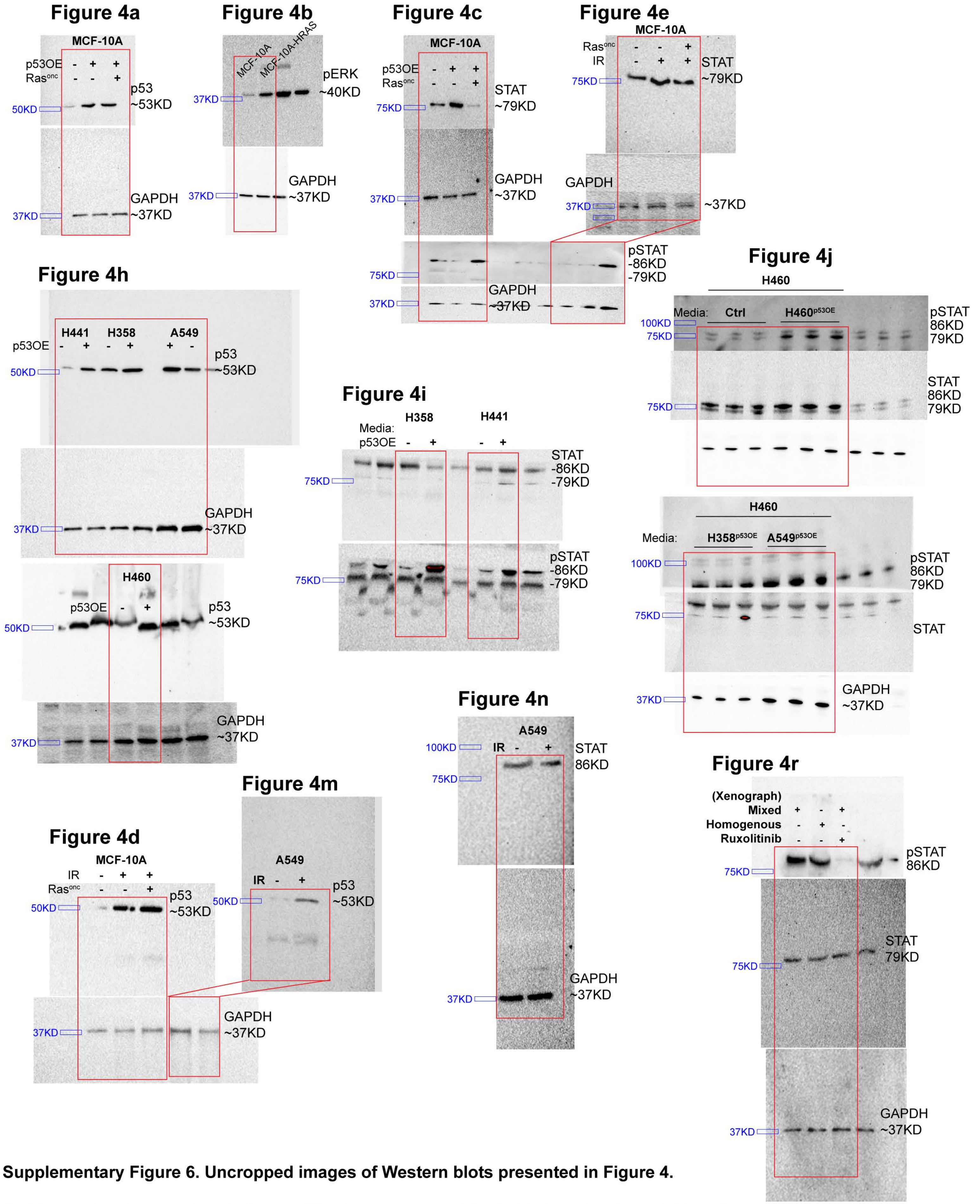

Supplementary Figure 6. Uncropped images of Western blots presented in Figure 4.

Red boxes denote area show in figures. Blue boxes represent the ladder's molecular weight markers.
