# Supplementary material for: Cooperation between oncogenic Ras and wild-type p53 stimulates STAT non-cell autonomously to promote tumor radioresistance
Source: Commun Biol. 2021 Mar 19;4:374. doi: 10.1038/s42003-021-01898-5 (PMC7979758; doi:10.1038/s42003-021-01898-5)
Supplement: Supplementary file 3 — Description of Additional Supplementary Files [file 42003_2021_1898_MOESM3_ESM.pdf]

Description of Additional Supplementary Files

File name: Supplementary Data 1

Description: Source data file for all graphs presented in the paper.
